# *Thismia hongkongensis* (Thismiaceae): a new mycoheterotrophic species from Hong Kong, China, with observations on floral visitors and seed dispersal

**DOI:** 10.3897/phytokeys.46.8963

**Published:** 2015-02-04

**Authors:** Shek Shing Mar, Richard M.K. Saunders

**Affiliations:** 1 Ying Wa College, 1 Ying Wa Street, Sham Shui Po, Hong Kong, P. R. China; 2 School of Biological Sciences, The University of Hong Kong, Pokfulam Road, Hong Kong, P. R. China

**Keywords:** Burmanniaceae, China, mycoheterotrophic, pollination, rain splash dispersal, *Thismia*, Thismiaceae, new species

## Abstract

A new species, *Thismia hongkongensis* S.S.Mar & R.M.K.Saunders, is described from Hong Kong. It is most closely related to *Thismia brunonis* Griff. from Myanmar, but differs in the number of flowers per inflorescence, the colour of the perianth tube, the length of the filaments, and the shape of the stigma lobes. We also provide inferences on the pollination ecology and seed dispersal of the new species, based on field observations and interpretations of morphology. The flowers are visited by fungus gnats (Myctophilidae or Sciaridae) and scuttle flies (Phoridae), which are likely to enter the perianth tube via the annulus below the filiform tepal appendages, and exit via small apertures between the filaments of the pendent stamens. The flowers are inferred to be protandrous, and flies visiting late-anthetic (pistillate-phase) flowers are possibly trapped within the flower, increasing chances of pollen deposition on the receptive stigma. The seeds are likely to be dispersed by rain splash.

## Introduction

*Thismia* Griff. species are small herbaceous plants with a highly reduced vegetative structure. They are invariably mycoheterotrophic, relying on fungal symbionts to obtain nourishment from decaying organic material, and they therefore lack chlorophyll. Individuals remain underground throughout most of the year, only emerging briefly to flower and fruit after periods of heavy rain; as a consequence, *Thismia* species are rarely collected and relatively little is known of their taxonomy, distribution and reproductive biology.

The floral morphology of the genus is complex. The tepals are congenitally fused (Caddick et al. 2000) to form a perianth tube with apically free lobes that are arranged in two whorls of three. These tepal lobes are often morphologically very elaborate, sometimes apically coherent and forming a mitre or dome, and often adorned with elongated tentacles that are either free or united. Although the functional significance of these morphologically complex tepals is obscure, they presumably perform a role in pollinator attraction. The aperture at the apex of the perianth tube is surrounded by a prominent annulus. Each flower has six stamens, the lateral margins of which are often postgenitally connate with each other, forming a ring that is suspended from the annulus. Since the stamens are pendent, the abaxial surface faces the centre of the flower, and the adaxial surface, which bears the thecae, faces the inner surface of the perianth tube. The flowers are epigynous, with the fused carpels surrounded by an expansion of the receptacle (Caddick et al. 2000).

Although *Thismia* was historically classified in the tribe Thismieae Miers (Miers 1847) within the Burmanniaceae (e.g., Jonker 1938; Maas-van der Kamer 1998), recent molecular phylogenetic analysis of nuclear and mitochondrial DNA sequences have revealed that it is more closely aligned with the Taccaceae (Merckx et al. 2006, 2009). The third iteration of the Angiosperm Phylogeny Group classification (APG 2009) adopts a very conservative approach in which *Thismia* is retained within the Burmanniaceae; we follow the majority of contemporary taxonomists, however, in separating *Thismia* and related genera (*Afrothismia* Schltr., *Haplothismia* Airy Shaw and *Oxygyne* Schltr.) in the Thismiaceae.

Govaerts et al. (2007) listed 41 *Thismia* species in their global checklist of the Dioscoreales, of which 24 were recorded from Asia; these statistics increase to 42 and 25, respectively, if *Geomitra clavigera* Becc. is recognized as a *Thismia* species, as suggested by morphological data (Stone 1980) and molecular phylogenetic analysis (Merckx et al. 2006). The discovery of new species in the genus has accelerated significantly in recent years, with 10 new *Thismia* species described from Asia (Larsen and Averyanov 2007; Chantanaorrapint 2008, 2012; Chiang and Hsieh 2011; Tsukaya and Okada 2012; Dančák et al. 2013; Li and Bi 2013; Nuraliev et al. 2014; Truòng et al. 2014) since the publication of the checklist by Govaerts et al. (2007). In addition to these newly described species, there are several reports of significant extensions to distributional ranges in Asia, including *Thismia alba* Holttum ex Jonker (Peninsular Malaysia and Peninsular Thailand: Chantanaorrapint and Sridith 2007), *Thismia clavigera* (Becc.) F.Muell. (Borneo, Langkawi, Sumatra and Peninsular Thailand: Stone 1980; Chantanaorrapint and Chantanaorrapint 2009), and *Thismia tentaculata* K.Larsen & Aver. (Vietnam and southern China: Ho et al. 2009).

In this paper we describe a new species, *Thismia hongkongensis*, recently collected from Hong Kong. This is the fourth species in the genus recorded from China, supplementing earlier reports of *Thismia taiwanensis* S.Z.Yang, R.M.K.Saunders & C.J.Hsu from Taiwan (Yang et al. 2002; Wu et al. 2010), *Thismia tentaculata* from Hong Kong (Ho et al. 2009; Wu et al. 2010; Zhang and Saunders 2011), and *Thismia gongshanensis* H.Q.Li & Y.K.Bi from Yunnan (Li and Bi 2013). We also present some new observational data and inferences on the pollination ecology and seed dispersal mechanism of the new species. Although this information is limited in scope, it is of significance given the paucity of existing data on the reproductive biology of the genus.

## New species description

### Thismia hongkongensis

Taxon classificationPlantaeDioscorealesThismiaceae

S.S.Mar & R.M.K.Saunders sp. nov.

urn:lsid:ipni.org:names:77145069-1

Figs 1 , 2 , 3 , 4

#### Diagnosis.

Similar to *Thismia brunonis* Griff., but differing in bearing a maximum of only three flowers (with a single flower at anthesis), and having a dark red perianth tube with the filiform appendages on the outer tepals remaining upright and forming a loose mitre over the annulus, longer staminal filaments with two distinct teeth at connective apex, and rounded stigmas.

#### Type.

**China:** 22°25'N, 114°11'E, Tai Po Kau Nature Reserve, New Territories, Hong Kong, 19 May 2014, *S.S. Mar 1* (holotype: HK, in spirit).

#### Description.

Small achlorophyllous holomycotrophic herbs. Roots clustered, ± horizontal, vermiform, fleshy, 1.2–1.3 mm in diameter, cream-coloured. Stem cream-coloured, unbranched, erect, ca. 1.7 cm tall, 1.8–2 mm in diameter, glabrous, terete, with ca. 12 longitudinal ridges. Leaves white, appressed, clasping stem, narrowly triangular with acute apex, scale-like, 3–5.5 mm long, 1.7–2 mm wide; basal leaves smallest, upper leaves (equivalent to floral bracts) largest. Flowers in clusters of up to 3, developing sequentially with only one anthetic. Perianth actinomorphic, of 6 fused tepals, forming a perianth tube with free apical lobes. Perianth tube pinkish-white, membranous, urceolate, ca. 6.7 mm long, ca. 6.1 mm in diameter, with 12 dark red vertical ribs, abaxial surface distinctly verrucose; apex of perianth tube fused to form a dark red, rounded-hexagonal annulus, ca. 1.4 mm wide (top, externally), ca. 2.3 mm wide (base, externally) and ca. 1 mm (internal aperture); dark red, inverted V-shaped structures (putative nectaries) at apex of adaxial surface of perianth tube, opposite apertures between staminal filaments. Outer tepal lobes triangular, ca. 1.8 mm long, ca. 1.5 mm wide at base; inner tepal lobes spathulate, concave adaxially, ca. 3.3 mm long, ca. 1.7 mm wide at widest point, bearing a dark red filiform appendage on the abaxial surface, ca. 4 mm long, ca. 0.5 mm in diameter (towards base), 0.4 mm in diameter (towards apex); the three filiform appendages remain upright and cross each other, forming a persistent mitre. Stamens 6, pendent from the inner margin of perianth annulus, ca. 2.9 mm long, ca. 1.1 mm wide at widest point; filaments free, ca. 1 mm long; stamens laterally connate, forming an anther tube; individual stamens with two thecae (adaxial, dehiscing towards inner surface of perianth tube), ca. 0.7 mm long; apical connective of stamens ca. 1 mm long, with two distinct teeth, adorned with trichomes, ca. 0.5 mm long. Ovary inferior, obconical, ca. 2.7 mm long, ca. 4 mm wide towards apex; style ca. 0.6 mm long, ca. 0.6 mm in diameter, with three bilobed, rounded stigmas; stigmatic head ca. 1 mm long, ca. 1 mm in diameter. Fruit a capsule ca. 4 mm long, ca. 4.8 mm wide, cup-shaped, carnose, pale orange-brown, dehiscing apically; fruiting peduncle ca. 2.5 mm diameter. Seeds numerous, yellow-brown.

#### Phenology.

**Figure 1.**
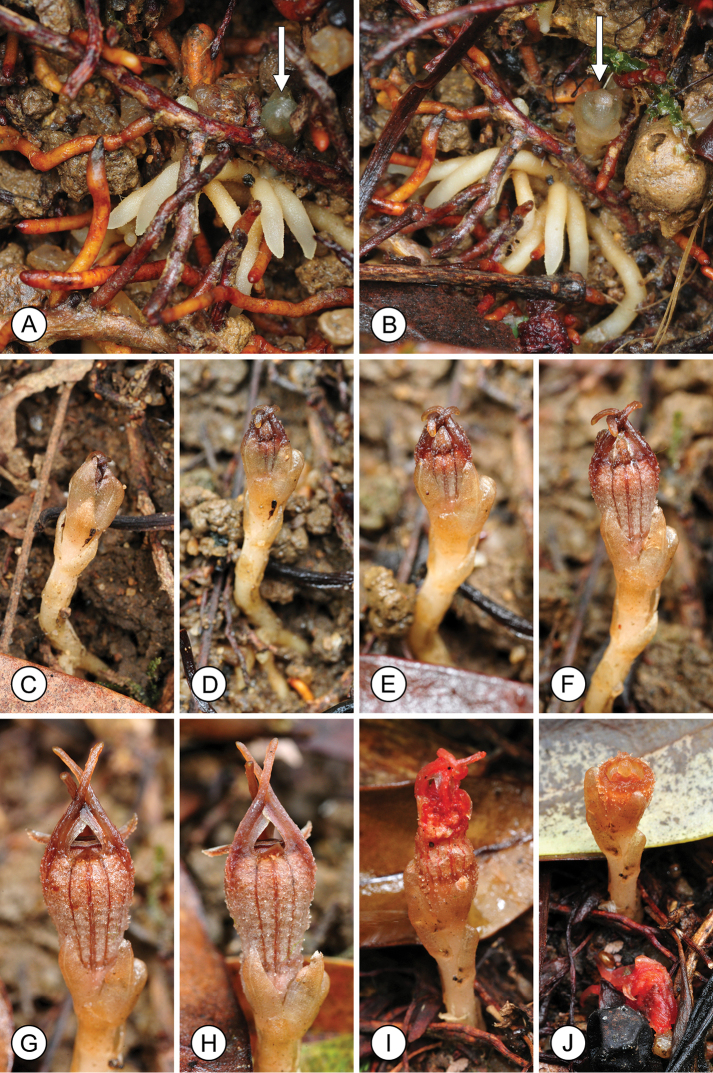
Flower development in *Thismia hongkongensis* sp. nov. **A, B** Root system, with young flowering stalk developing (arrowed). **C–H** Developing flower, photographed over a 17-day period (10^th^, 14^th^, 16^th^, 19^th^, 23^rd^ and 27^th^ May, respectively) (*S.S. Mar 1*, HK). **I, J** Post-fertilization flower, showing abscission of perianth tube. Photos by S.S. Mar.

**Figure 2.**
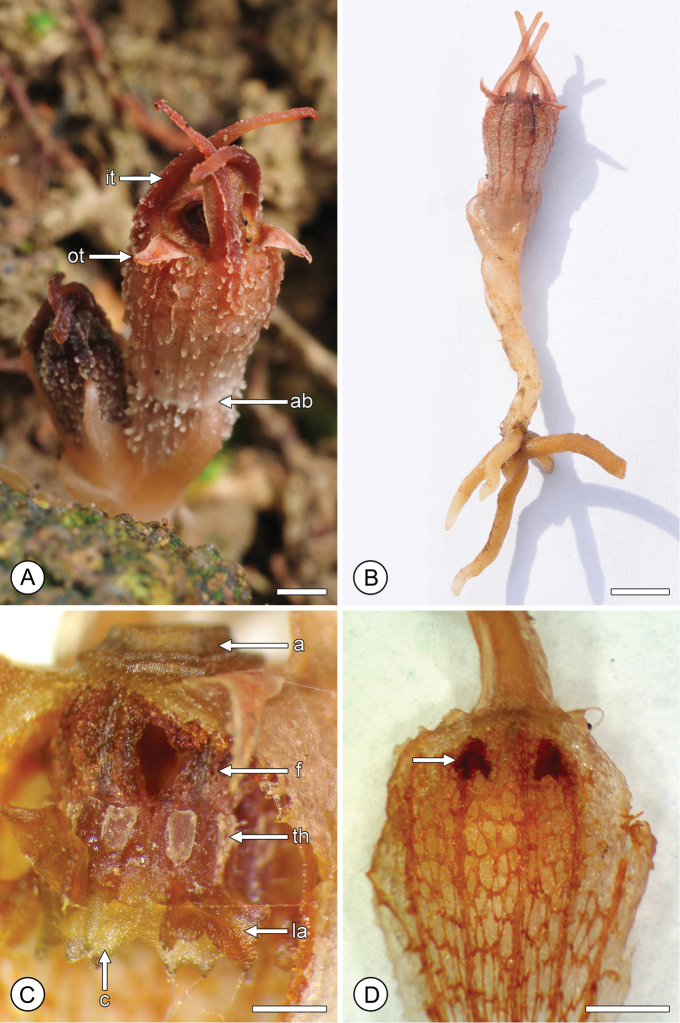
Flower structure in *Thismia hongkongensis* sp. nov. **A** Mature flower, showing outer tepals (ot), inner tepals (it) and abscission zone (ab) at the base of the perianth tube. **B** Entire plant (*S.S. Mar 1*, HK). **C** Perianth tube with annulus (a), following removal of the proximal face of the tube, exposing pendent stamens with filament (f), thecae (th), connective (c) and lateral appendage (la) (*S.S. Mar 2*, HK). **D** Inner face of perianth tube, showing network patterning and putative nectaries (arrowed) (*S.S. Mar 2*, HK). Scale bars: **A, D** = 2 mm; **B** = 5 mm; **C** = 1 mm. Photos: **A, B** S.S. Mar; **C, D** R.M.K. Saunders.

**Figure 3.**
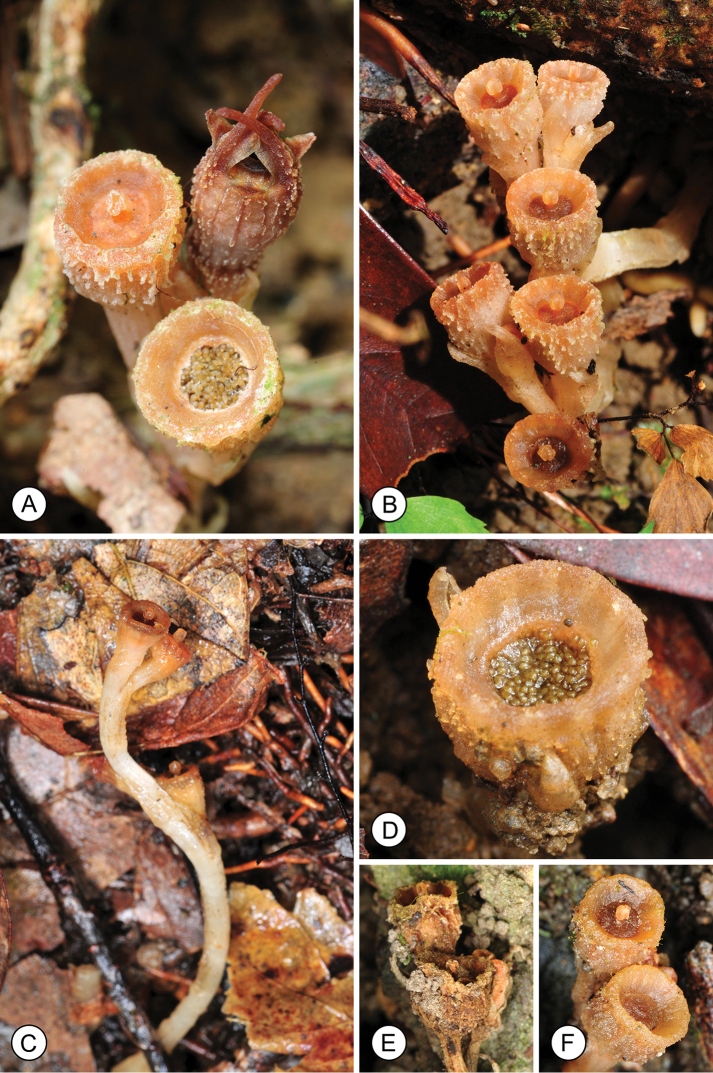
Fruit structure in *Thismia hongkongensis* sp. nov. **A** Flower (rear right), immature fruit, shortly after fertilization (left), and mature fruit with exposed seeds (front). **B** Two fruiting individuals, each with three fruits. **C** Lateral view of fruiting specimen, illustrating elongated fruit stalk. **D** Mature fruit with exposed seeds. **E** Dehydrated fruit. **F** Rehydrated fruit, after rainfall. Photos by S.S. Mar.

Flowering was observed between May and September. Flower development extends over several weeks (Fig. 1C–H). Based on our field observations, mature flowers are functional for up to three weeks, with up to three flowers developing successively in each individual (Fig. 3A). The perianth tube abscises after fertilization (Fig. 1I, J), with a clear abscission zone (*ab* in Fig. 2A); the epidermis on the upper surface of the carpel subsequently disintegrates and the stigma is shed, exposing the seeds (Fig. 3A, D). Fruiting was observed between June and October.

#### Distribution and habitat.

*Thismia hongkongensis* is only known from the type locality in Hong Kong. The habitat is lowland secondary forest (west-facing slope, ca. 240 m above sea level); the site is not close to a stream and is therefore likely to dry out during periods of low precipitation. Cooccurring species include *Adiantum flabellulatum* L. (Adiantaceae), *Ardisia quinquegona* Blume (Myrsinaceae), *Breynia fruticosa* (L.) Hook.f. (Euphorbiaceae), *Burmannia wallichii* (Miers) Hook.f. (Burmanniaceae), *Clematis meyeniana* Walp. (Ranunculaceae), *Cratoxylum cochinchinense* (Lour.) Blume (Clusiaceae), *Desmos chinensis* Lour. (Annonaceae), *Diplospora dubia* (Lindl.) Masam. (Rubiaceae), *Garcinia oblongifolia* Champ. ex Benth. (Clusiaceae), *Lindsaea orbiculata* (Lam.) Mett. ex Kuhn (Lindsaeaceae), *Lophatherum gracile* Brogn. (Poaceae), *Lygodium japonicum* (Thunb.) Sw. (Lygodiaceae), *Machilus chekiangensis* S.K.Lee (Lauraceae), *Psychotria asiatica* L. (Rubiaceae), *Psychotria serpens* L. (Rubiaceae), *Rourea microphylla* (Hook. & Arn.) Planch. (Connaraceae), *Sarcandra glabra* (Thunb.) Nakai (Chloranthaceae), and *Sciaphila ramosa* Fukuy. & T.Suzuki (Triuridaceae).

#### Etymology.

The specific epithet reflects the geographical origin of the species in Hong Kong.

#### Additional specimens examined

**(paratypes).** China. Hong Kong: 22°25'N, 114°11'E, Tai Po Kau Nature Reserve, New Territories, 29 May 2014, *S.S. Mar 2* (HK, dissected flower in spirit); idem, 2 October 2014, *S.S. Mar 3* (HK, immature fruit in spirit).

#### Discussion.

**Figure 4.**
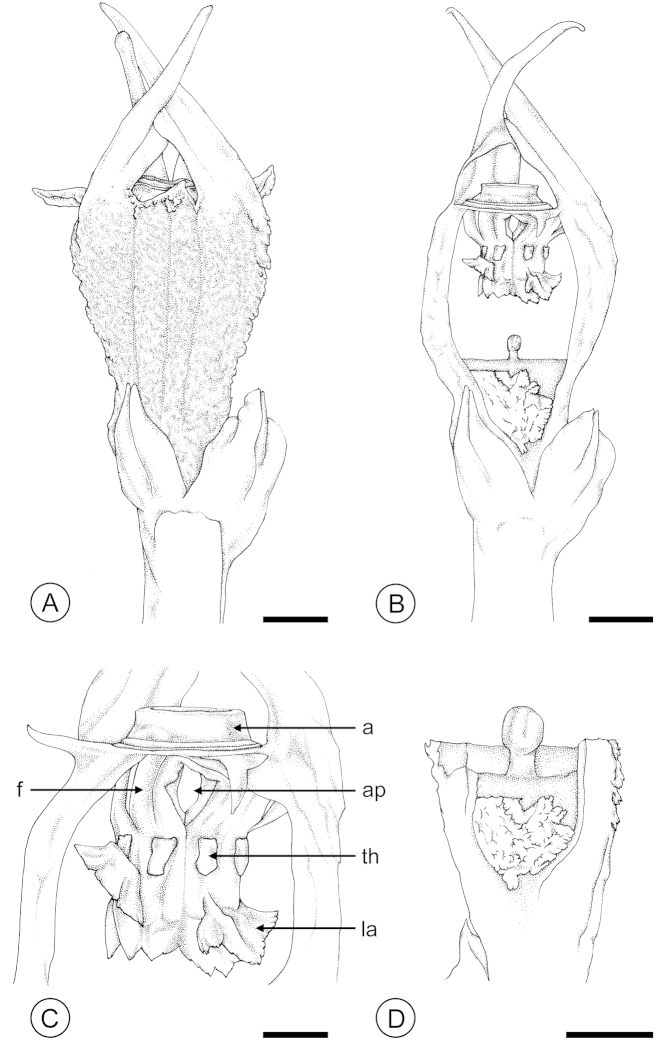
*Thismia hongkongensis* sp. nov. (*S.S. Mar 2*, HK). **A** Entire flower. **B** Flower with proximal part of perianth tube removed, showing pendent stamens. **C** Apex of the perianth tube, showing annulus (a) and pendent stamens, with filament (f), thecae (th), lateral appendage (la), and aperture (ap) between filaments. **D** Longitudinal section through fused carpels. Scale bars: **A, B, D** = 2 mm; **C** = 1 mm. Drawings by Caren Pearl Shin.

*Thismia hongkongensis* is most similar to *Thismia brunonis* Griff. (1844, 1845; as ‘*Thismia brunoniana*’ in the latter), the type species in the genus. *Thismia brunonis* is only known from a single collection from Tenasserim in Myanmar, dating from October 1834. According to Jonker’s (1938) supraspecific classification of the genus, both species would be included in sect. *Thismia* (‘*Euthismia*’) subsect. *Brunonithismia* Jonker as they have unequal and free tepal lobes. Comparison of the two species reveals several significant differences, however, including flower number per inflorescence. The protologue of *Thismia brunonis* includes reference to flowers clustered in a terminal raceme (“Flores pauci, in racemum brevem terminalem dispositi”: Griffith 1845: 341) with the accompanying illustration in the same publication (Fig. 1 in Pl. XXXIX) showing inflorescences with four and six flowers; Jonker (1938) subsequently described the species as bearing 3–8 flowers per raceme. In contrast, *Thismia hongkongensis* invariably has fewer flowers, with a maximum of three per inflorescence, reaching anthesis consecutively. The perianth tube of *Thismia brunonis* is pale yellow with red ribs (Griffith 1845), whilst it is pink with red ribs in *Thismia hongkongensis* (Figs 1G–J, 2A, B). The filiform appendages on the outer tepals of *Thismia brunonis* appear to be spreading at maturity (Griffith 1845: Pl. XXXIX), whereas those of *Thismia hongkongensis* invariably remain upright and cross each other to form a loose mitre over the annulus (Figs 1G–J, 2A, B, 4A). *Thismia brunonis* also differs from *Thismia hongkongensis* as it has a much shorter filament: although a measurement was not included in Griffith’s descriptions of *Thismia brunonis*, the accompanying plate (Griffith 1845: Fig. 7 in Pl. XXXIX) indicates that it is considerably shorter than the rest of the stamen. The shape of the stigma lobes also differs: it is acute in *Thismia brunonis* (Griffith 1845: Fig. 9 in Pl. XXXIX; Jonker 1938), but rounded in *Thismia hongkongensis* (Fig. 4D).

*Thismia hongkongensis* is strikingly different from its congener in Hong Kong. *Thismia tentaculata* has a white perianth tube with a bright yellow annulus, and three divergent orange-red filiform appendages on the inner tepals (Ho et al. 2009).

#### IUCN conservation status.

CR D (IUCN 2001). Only one population is known, consisting of ca. 10 individuals, covering an area of approximately 4–5 m^2^. The population is located within the Tai Po Kau Nature Reserve, but is close to a major hiking path and the population is therefore susceptible to human disturbance and vegetation clearance.

##### Pollination ecology

Although the Burmanniaceae s.l. (inclusive of *Thismia*) are reported to be protandrous (Vogel 1998), this inference was based on a paraphyletic circumscription of the family and it is unclear whether protandry has specifically been observed in *Thismia*. Dissections of late-anthetic flowers of *Thismia hongkongensis* allow tentative confirmation of protandry, however, as the thecae are completely devoid of pollen (*th* in Fig. 2C).

Little is known of the pollinators of *Thismia*, although several authors have suggested that the flowers may be visited by small flies (Vogel 1962; Stone 1980; Rübsamen 1986). These inferences were based on perianth morphology, the presence of osmophores on the tepals (Vogel 1962), the presence of nectaries either at the base of the perianth (Poulsen 1890) or along the suture between contiguous anthers (e.g., Groom 1895; Pfeiffer 1918; Jonker 1938, 1948; Cribb 1986, 1995; Thiele and Jordan 2002; Ho et al. 2009), and the formation of sticky pollen (Cranwell 1953). The only previous observational report of insect visitors to *Thismia* flowers is of small, unidentified flies entering the perianth tube of *Thismia gongshanensis* (Li & Bi, 2013).

Several researchers have inferred that fungus gnats are likely to pollinate *Thismia* flowers based on structural mimicry (e.g., tepal extensions and reticulate patterning on the inner surface of the perianth tube), perianth colour and the emission of fungus-like floral scents (Vogel 1978; Rübsamen 1986; Thiele and Jordan 2002). We retrieved a fungus gnat (belonging to either the Mycetophilidae or Sciaridae) from within the perianth tube of a late-anthetic specimen of *Thismia hongkongensis*; unfortunately the poor state of preservation of the fly precluded further identification. We also retrieved an isolated insect wing from within the floral chamber which had the characteristic venation typical of a scuttle fly (Phoridae). Fungus gnats and scuttle flies are generally associated with decaying organic matter and are often reported to feed on fungi (Hill et al. 1982).

The pollinators presumably enter the floral chamber of *Thismia hongkongensis* via the aperture within the annulus (*a* in Figs 2C, 4C). Assuming that the flower is protandrous as suggested, the movements of the pollinators are likely to differ depending on whether the flower is in its early anthetic (staminate) or late anthetic (pistillate) phase. In staminate-phase flowers, the flies are likely to be attracted to the putative nectaries (arrowed in Fig. 2D) located at the apex of the perianth tube, behind the pendent staminal ring. We hypothesize that the irregular surface on the adaxial surface of the perianth tube resulting from the reticulate venation (Fig. 2D) possibly enables the insects to climb and access these nectaries. The flies are likely to make contact with the thecae (*th* in Figs 2C, 4C) and inadvertently collect pollen whilst feeding from the nectaries, before departing via the small apertures (ca. 0.5 mm diameter) located between the filaments (*f* in Figs 2C, 4C) of adjacent stamens. If the flies enter late-anthetic pistillate-phase flowers, however, it is possible that they might be prevented from accessing the nectaries because of the raised lateral appendages of the stamens (*la* in Figs 2C, 4C), thereby increasing the time in which contact with the stigma is possible. The possible trapping of flies may explain the frequency with which flies are observed inside the perianth tube of late-anthetic flowers. Similar interpretations of pollinator movement, involving climbing the inner wall of the perianth tube and exiting via the apertures between the filaments, has previously been suggested by Maas et al. (1986) and Thiele and Jordan (2002) for other *Thismia* species.

##### Seed dispersal

Several different interpretations of seed dispersal have been proposed for *Thismia*, including movement by earthworms with secondary dispersal possible if the worms are eaten by birds (Beccari 1890). Stone (1980) suggested that *Thismia* seeds are likely to be dispersed by raindrops that splash seeds out of the fruit-cup. The size and shape of the fruit-cups of *Thismia hongkongensis* closely resemble functionally similar rain-splash dispersal structures in phylogenetically disparate groups (Nakanishi 2002): the upper surface of the fruit disintegrates at maturity (Fig. 3A), resulting in an upright, cup-like hypanthium (ca. 4.8 mm in diameter) with seeds that are fully exposed (Fig. 3D). Studies of other plant groups indicate dispersal of up to 1 m (Nakanishi 2002), although there are inevitably many variables (including plant height, seed weight, etc.) that affect potential dispersal distance; it is perhaps significant that the stem of *Thismia hongkongensis* elongates as the fruit matures (Fig. 3C), thereby possibly increasing seed dispersal distance.

Brodie (1951) observed that seeds of species that are rain-splash dispersed are often coated with a hydrophilic colloid that rapidly absorbs water, and which may act as a lubricant, facilitating separation of seeds by rain drops. *Thismia hongkongensis* seeds are surrounded by a conspicuous mucilage-like substance (Fig. 3D) that may function in this way.

The fruits of *Thismia hongkongensis* appear to remain functional for several weeks. Although the plants are inevitably subjected to periods of desiccation, the fruits appear to be able to rehydrate effectively (Fig. 3E, F), thereby prolonging the period for seed dispersal.

## Supplementary Material

XML Treatment for Thismia hongkongensis
